# Starvation Induces Extracellular Accumulation of Polyphosphate in *Dictyostelium discoideum* to Inhibit Macropinocytosis, Phagocytosis, and Exocytosis

**DOI:** 10.3390/ijms24065923

**Published:** 2023-03-21

**Authors:** Ramesh Rijal, Issam Ismail, Shiyu Jing, Richard H. Gomer

**Affiliations:** Department of Biology, Texas A&M University, College Station, TX 77843-3474, USA

**Keywords:** polyphosphate, *Dictyostelium*, development, membrane fluidity, nutrients, cell mass

## Abstract

*Dictyostelium discoideum* is a soil-dwelling unicellular eukaryote that accumulates extracellular polyphosphate (polyP). At high cell densities, when the cells are about to overgrow their food supply and starve, the corresponding high extracellular concentrations of polyP allow the cells to preemptively anticipate starvation, inhibit proliferation, and prime themselves to begin development. In this report, we show that starved *D. discoideum* cells accumulate cell surface and extracellular polyP. Starvation reduces macropinocytosis, exocytosis, and phagocytosis, and we find that these effects require the G protein-coupled polyP receptor (GrlD) and two enzymes, Polyphosphate kinase 1 (Ppk1), which is required for synthesizing intracellular polyP, cell surface polyP, and some of the extracellular polyP, and Inositol hexakisphosphate kinase (I6kA), which is required for cell surface polyP and polyP binding to cells, and some of the extracellular polyP. PolyP reduces membrane fluidity, and we find that starvation reduces membrane fluidity; this effect requires GrlD and Ppk1, but not I6kA. Together, these data suggest that in starved cells, extracellular polyP decreases membrane fluidity, possibly as a protective measure. In the starved cells, sensing polyP appears to decrease energy expenditure from ingestion, and decrease exocytosis, and to both decrease energy expenditures and retain nutrients.

## 1. Introduction

Eukaryotic cells possess the ability to uptake particles by phagocytosis or fluid by macropinocytosis. Phagocytosis and macropinocytosis share a common evolutionary origin, and these processes co-evolved as feeding mechanisms [1]. *Dictyostelium discoideum* is a soil amoeba that feeds on bacteria by phagocytosis [1,2,3]. Some *D. discoideum* strains can be grown axenically in a defined liquid nutrient medium, and these axenic strains possess an increased rate of macropinocytosis [4,5,6,7,8,9]. The axenic strains have a mutation in the gene encoding the Ras GTPase activating the protein Neurofibromin (NF1) that regulates both phagocytosis and macropinocytosis [10], and the loss of NF1 potentiates Ras activation at the sites where membrane ruffles form during macropinocytosis, thus increasing macropinocytosis [10]. Following endocytosis, the particle or fluid is transferred to lysosomes where the particle or fluid is digested, and the undigested material is then exocytosed [11,12].

In nutrient-rich conditions, *D. discoideum* exists as unicellular amoebae. Starvation initiates a developmental cycle where cells aggregate together to form a multicellular fruiting body consisting of a mass of spores held off the substrate by a column of stalk cells [13,14]. During development, starting 1 h after starvation, *D. discoideum* cells suppress macropinocytosis by ~80% [15], and after 6 h of starvation, *D. discoideum* cells suppress phagocytosis by ~50% [16]. At 8 h of starvation, most *D. discoideum* cells have almost completely reduced both macropinocytosis and phagocytosis [16]. However, during development, a small percentage of cells maintain high levels of phagocytosis and behave similar to immune cells that patrol the multicellular structures [17,18]. In addition, starting 1 h after starvation, *D. discoideum* cells reduce exocytosis, and at 4 h exocytosis is reduced by >50% [15]. How *D. discoideum* cells inhibit macropinocytosis, phagocytosis, and exocytosis during development remains unclear.

Polyphosphate (polyP) is a polymer of inorganic phosphate residues linked by phosphoanhydride bonds. PolyP is present in all life kingdoms [19], and it is involved in energy and phosphate storage, survival under stress conditions, and biofilm formation, and virulence in prokaryotes, and blood coagulation, inflammation, proliferation of leukemia cells, and bone calcification in eukaryotes [19,20,21,22,23,24,25,26]. Proliferating *D. discoideum* cells continuously secrete polyP, and when the cells have reached a high cell density where the cells will be about to overgrow their nutrient source, the corresponding high extracellular polyP concentrations inhibit proliferation, reduce phagocytosis, macropinocytosis, and exocytosis, and induce aggregation [24,27,28]. *D. discoideum* synthesizes some polyP using polyphosphate kinase 1 (Ppk1), a highly conserved enzyme in prokaryotes, and likely to have been acquired by *D. discoideum* through horizontal gene transfer [29,30]. *D. discoideum* cells lacking Ppk1 have undetectable levels of intracellular polyP [30], and reduced but detectable levels of extracellular polyP [27]. PolyP levels are also reduced in cells lacking inositol hexakisphosphate kinase A (I6kA), an enzyme that phosphorylates the inositol pyrophosphate IP6 to generate IP7 [27,31]. This suggests that both Ppk1 and I6kA are involved in polyP synthesis in *D. discoideum* and either of the enzymes might compensate for the loss of the other enzyme. PolyP shows saturable binding to *D. discoideum,* and the binding is lost in cells lacking the G protein-coupled receptor GrlD [32]. GrlD is required for polyP inhibition of proliferation [32]. In addition, GrlD, Ppk1, and I6kA are all required for polyP inhibition of macropinocytosis and exocytosis in proliferating cells [27,28]. Although polyP inhibits phagocytosis in proliferating cells [28], we do not know whether GrlD, Ppk1, or I6kA are required for this inhibition.

PolyP induces aggregation of *D. discoideum* cells even in the presence of nutrients [24], suggesting that high levels of extracellular polyP may act as a signal that lets cells sense that they are at a high cell density, and thus probably about to overgrow their food supply and starve. This, in turn, would allow cells to be able to anticipate starvation and begin development. Throughout development, *D. discoideum* cells accumulate high concentrations of intracellular polyP, and the concentrations reach > 100-fold in the fruiting body compared to vegetative cells [30,33].

Little is known about the function of polyP during *D. discoideum* development. In this report, we examined how *D. discoideum* regulates polyP production in response to starvation. We found that after 6 h of starvation, *D. discoideum* accumulates large amounts of secreted and cell-surface extracellular polyP, and the development-associated reduction in macropinocytosis, exocytosis, and phagocytosis requires GrlD, Ppk1, and I6kA. This then suggests that the development-associated reduction in macropinocytosis, exocytosis, and phagocytosis is caused by high extracellular levels of polyP sensed by the receptor GrlD.

## 2. Results

### 2.1. Starving D. discoideum Cells Require GrlD and Ppk1 to Accumulate Normal Levels of Extracellular and Cell Surface polyP, and I6kA to Accumulate Normal Levels of Cell Surface polyP

**Figure 1:**
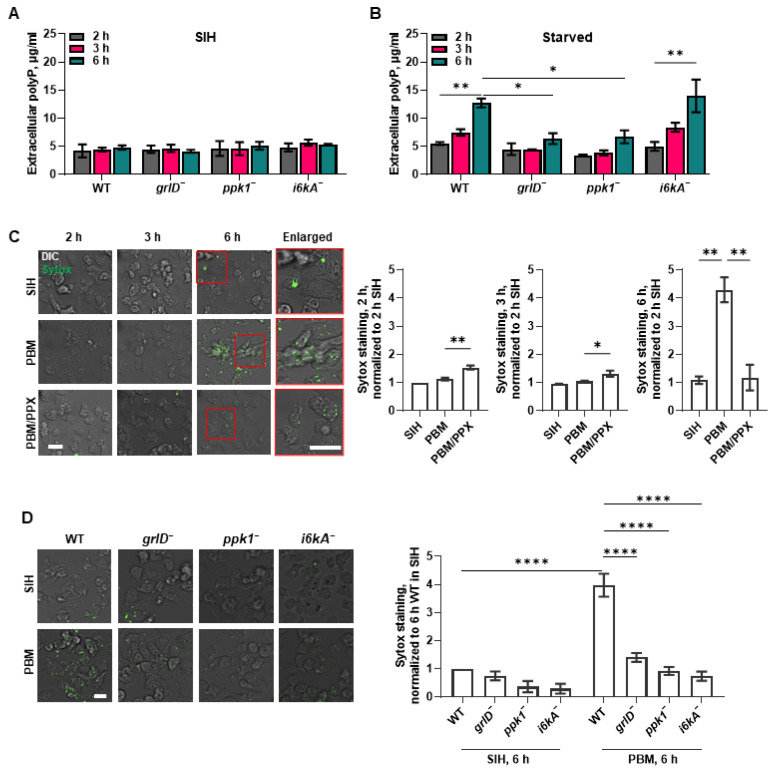
Starving *D. discoideum* cells require GrlD and Ppk1 to accumulate extracellular polyP, and GrlD, Ppk1, and I6kA to accumulate cell surface polyP. (**A**,**B**) Wild-type (WT), *grlD^−^*, *ppk1^−^*, and *i6kA^−^* cells were incubated in SIH (**A**) or starved in PBM (**B**) for 2, 3, and 6 h and extracellular polyP concentrations were measured at the indicated times. (**C**) WT cells were incubated in SIH, PBM, or PBM with PPX for 2, 3, and 6 h, stained with SYTOX (green), and the mean fluorescence intensities of SYTOX staining were measured at the indicated times. The mean fluorescence intensity of SYTOX staining in cells incubated in SIH for 2 h was considered 1. Representative images of 3 independent experiments are shown. Cell boundaries in the enlarged insets show the SYTOX staining (green) of cells. DIC indicates differential interference contrast. Bars are 10 µm. (**D**) WT, *grlD^−^*, *ppk1^−^*, and *i6kA^−^* cells were incubated in SIH or PBM for 6 h, stained with SYTOX, and the mean fluorescence intensities of SYTOX staining were measured. The mean fluorescence intensity of SYTOX staining in cells incubated in SIH for 6 h was considered 1. Representative images of 3 independent experiments are shown. Bar is 10 µm. Values are mean ± SEM of 3 independent experiments (**A**–**D**). * indicates *p* < 0.05, ** *p* < 0.01, and **** *p* < 0.0001 (One-way ANOVA with Dunnett’s multiple comparisons test (**B**,**C**)) (Two-way ANOVA with Bonferroni’s multiple comparisons test (**D**)).

Proliferating *D. discoideum* cells accumulate extracellular polyP, and the extracellular polyP concentration reaches a maximum (~705 µg/mL) when the cell density reaches a maximum (stationary phase; ~2 × 10^7^ cells/ml) [27]. Loss of either Ppk1 or I6kA reduces this accumulation by ~50%, although only at very high cell densities [27]. The cells then require GrlD to sense the extracellular polyP [32]. Starvation induces the accumulation of intracellular polyP in *D. discoideum* cells [30,33]. To determine if *D. discoideum* cells accumulate extracellular polyP during starvation, wild-type (WT) cells or cells lacking GrlD (*grlD^−^*), polyphosphate kinase 1 (*ppk1^−^*), or inositol hexakisphosphate kinase A (*i6kA^−^*) were incubated for 6 h in the defined growth medium SIH, or the starvation buffer PBM. Conditioned medium (CM) was collected and clarified, and the polyP concentration was quantified by incubating CM with DAPI and measuring fluorescence at 550 nm after excitation at 415 nm [28,34]. As previously observed [27], mid-log phase WT, *grlD^−^*, *ppk1^−^*, and *i6kA^−^* cells had comparable levels of extracellular polyP (Figure 1A). Compared with cells in SIH or cells starved for 2 h (Figure 1A,B), extracellular polyP levels in WT cells increased after 6 h in PBM (Figure 1B). Compared to WT cells, *grlD^−^* and *ppk1^−^* cells accumulated less extracellular polyP after starvation for 6 h (Figure 1B). The *i6kA^−^* cells had extracellular polyP concentrations similar to WT (Figure 1A,B). Together, these data suggest that *D. discoideum* cells accumulate extracellular polyP during the early stages of starvation, that I6kA is not necessary for this effect, and that Ppk1 mediates some but not all of the extracellular polyP accumulation. It is likely that because sensing polyP is needed for cells to accumulate polyP, cells also require GrlD to increase extracellular polyP at 6 h of starvation.

**Figure 2:**
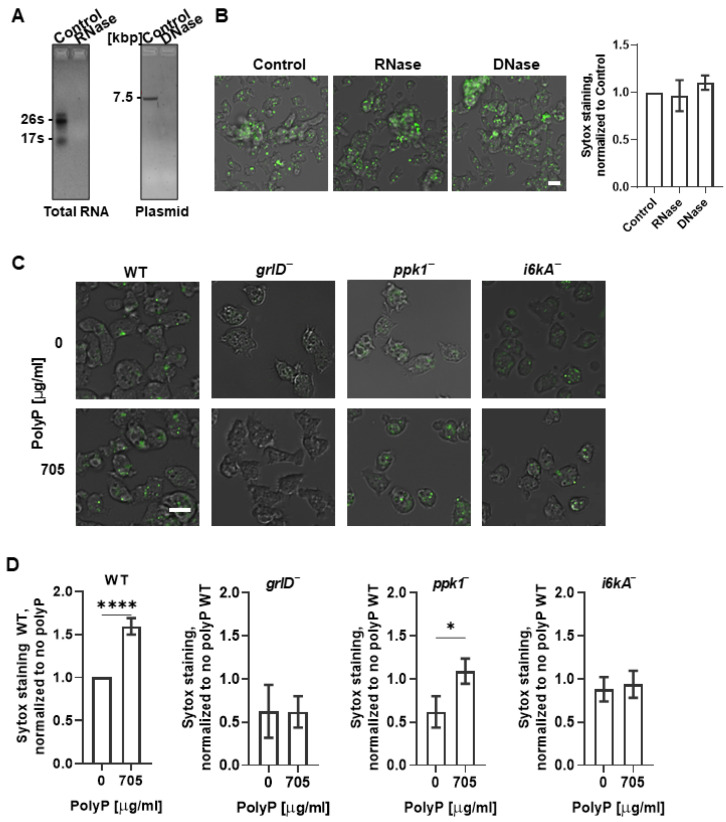
Starving *D. discoideum* cells require GrlD and I6kA to bind exogenous polyP. (**A**) Agarose gels of total RNA or plasmid DNA untreated (Control) or treated with RNase or DNase, respectively, stained with ethidium bromide are shown. Kbp indicates kilo base pair, and s indicates Svedbergs, a unit for sedimentation coefficient. (**B**) WT cells starved in PBM for 6 h were treated with RNase or DNase and stained with SYTOX, and the normalized mean fluorescence intensities of SYTOX staining were determined. (**C**) Vegetative WT, *grlD^−^*, *ppk1^−^*, and *i6kA^−^* cells were incubated no polyP (0) or with 705 µg/mL polyP and stained with SYOTX. For (**B**,**C**), representative merged images of differential interference contrast (DIC) and the SYTOX staining (green) are shown from 3 independent experiments. Bars are 10 µm. (**D**) Quantification of the mean fluorescence intensities of SYTOX staining from C are shown. The mean fluorescence intensity of the SYTOX staining in WT cells with no polyP (0) was considered 1. Values in (**B**,**D**) are mean ± SEM of 3 independent experiments. * indicates *p* < 0.05 and **** *p* < 0.0001 (*t* tests).

PolyP forms condensed spherical nanoparticles on the surface of activated human platelets [35]. Cell-surface-associated polyP can be detected with the cell-impermeable high affinity nucleic acid stain SYTOX [35]. To determine if extracellular polyP is present on the cell surface, cells were stained with SYTOX. We found that WT cells starved for 6 h showed increased SYTOX staining compared to cells in SIH (Figure 1C,D). To determine if the SYTOX staining was due to nucleic acid that is present on the cell surface, for instance due to release from dead cells, WT cells were starved in PBM for 5.5 h, then ribonuclease (RNase) or deoxyribonuclease (DNase) were added to cells, and the cells were incubated for 30 min, and then SYTOX staining of the cells was performed. We also tested the enzymatic activity of RNase and DNase by incubating RNA or DNA with RNase or DNase at the concentration that was used to treat the cells. At concentrations where the RNase or DNase completely digested RNA or DNA, respectively (Figure 2A), RNase or DNase did not significantly affect the fluorescence intensity of the SYTOX staining (Figure 2B), suggesting that the fluorescence on cells from SYTOX staining is not due to the presence of extraneous nucleic acids, but likely due to the presence of polyP.

In the control assays for Figure 1C,D, cells were incubated in PBM in the presence of 5 µg/mL of yeast exopolyphosphatase (PPX), an enzyme that degrades polyP by removing terminal phosphate residues [36]. At 2 and 3 h of incubation, the SYTOX staining was slightly higher in the PPX-treated group than in the cells incubated in SIH or PBM alone. It is possible that *D. discoideum* might compensate for an initial loss of polyP when exposed to PPX by synthesizing more polyP. However, in cells incubated in PBM with PPX at 6 h, the fluorescence intensity of the SYTOX staining was significantly lower than in the cells incubated in SIH or PBM alone (Figure 1C). This suggests that cells do not compensate for a persistent degradation of extracellular polyp or cannot maintain high levels of production, and that the SYTOX staining at 6 h of starvation may be due to polyP on the cells. In SIH, *ppk1*^−^ and *i6kA*^−^ appeared to have decreased SYTOX staining, but this was not statistically significant (Figure 1D). For cells starved for 6 h, compared to WT cells, *grlD*^−^, *ppk1*^−^, and *i6kA*^−^ cells showed decreased SYTOX staining (Figure 1D). These data suggest that in addition to the accumulation of extracellular polyP at 6 h of starvation, *D. discoideum* cells accumulate cell surface polyP at 6 h of starvation, and that this process is potentiated by GrlD, Ppk1, and I6kA.

### 2.2. Cells Require GrlD and I6kA to Bind Exogenous polyP

WT *D. discoideum* cells bind extracellular polyP, and the loss of GrlD reduces the binding of polyP to cells [27,32]. To test if the binding of extracellular polyP to WT cells increases the SYTOX staining, cells in SIH were incubated with exogenous polyP and incubated with SYTOX. As in Figure 1D, in SIH in the absence of exogenous polyP, WT, *grlD*^−^, *ppk1*^−^, and *i6kA*^−^ cells showed similar levels of SYTOX staining. Incubation with exogenous polyP increased the SYTOX staining of WT and *ppk1^−^* cells, but not *grlD^−^* or *i6kA^−^* cells (Figure 2C,D). Together, the data suggest that SYTOX shows a basal staining on cells, and that GrlD and I6kA are needed for cells to bind additional exogenous polyP.

### 2.3. Starvation Reduces the Cell Membrane Fluidity of D. discoideum Cells, and This Requires GrlD and Ppk1

**Figure 3:**
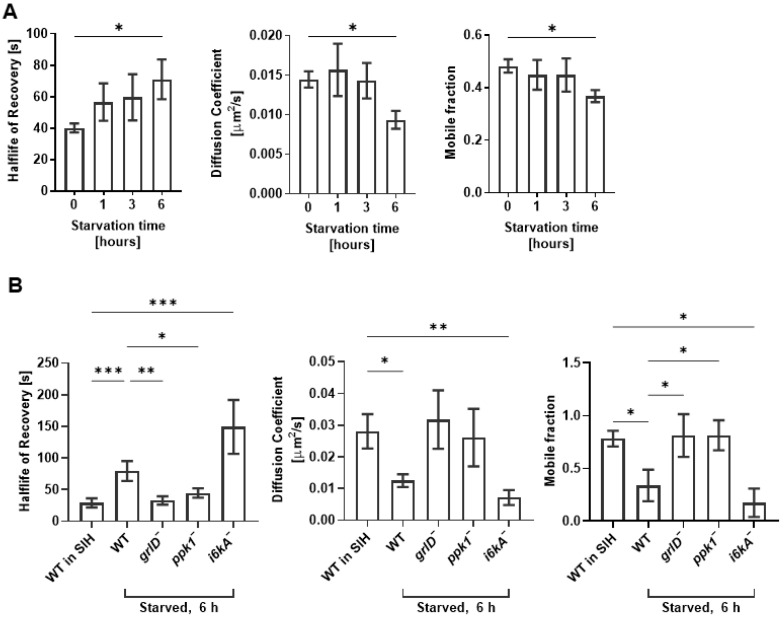
Starvation reduces cell membrane fluidity of WT and *i6kA^−^* cells. (**A**,**B**) Half-life of recovery, diffusion coefficient, and mobile fraction were calculated from fluorescence recovery after photobleaching of WT cells starved in PBM for 0, 1, 3 and 6 h (**A**) and WT cells incubated in SIH for 0 h or WT, *grlD^−^*, *ppk1^−^*, and *i6kA^−^* cells starved in PBM for 6 h (**B**). Values are mean ± SEM of 3 independent experiments. * *p* < 0.05, ** *p* < 0.01, *** *p* < 0.001 (One-way ANOVA with Brown-Forsythe and Welch test).

Cell membrane physical properties such as membrane fluidity are important regulators of endocytosis and exocytosis in mammalian cells [37,38]. We previously observed that in SIH (where cells are unstarved and vegetative), WT, *grlD^−^*, *ppk1^−^*, and *i6kA*^−^ cells have similar membrane fluidity, that polyP decreases membrane fluidity of WT cells, and that this requires GrlD, Ppk1, and I6kA [28]. Compared to WT and *i6kA*^−^ cells in SIH [28] or freshly starved WT cells, WT and *i6kA*^−^ cells starved for 6 h showed a decreased membrane fluidity as indicated by an increased half-life of recovery, decreased diffusion coefficient, and decreased mobile fraction (Figure 3A,B). Compared with cells in SIH ([28] and Figure 3B), the *grlD*^−^ and *ppk1*^−^ cells starved for 6 h did not show decreased membrane fluidity (Figure 3B). Together, these data suggest that starvation reduces cell membrane fluidity in *D. discoideum* cells, and that this effect requires GrlD and Ppk1 but not I6kA.

### 2.4. Starving D. discoideum Cells Require GrlD, Ppk1, and I6kA to Reduce Macropinocytosis and Nutrient Retention

**Figure 4:**
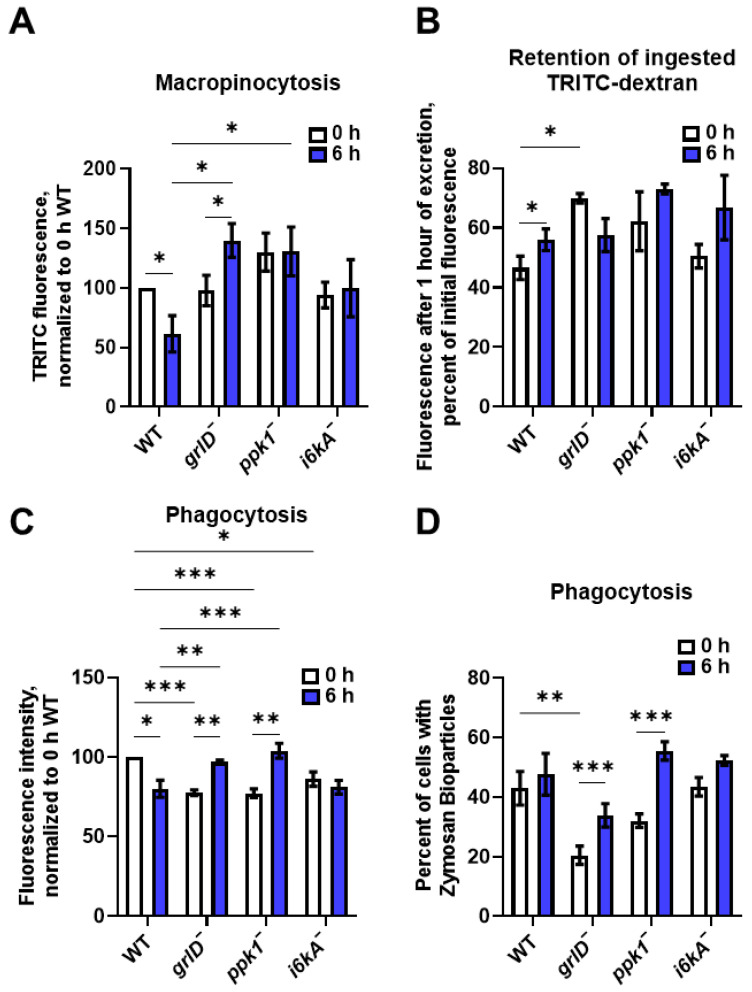
Starving *D. discoideum* cells require GrlD, Ppk1, and I6kA to reduce macropinocytosis, exocytosis, and phagocytosis. (**A**) WT, *grlD^−^*, *ppk1^−^*, and *i6kA^−^* cells starved in PBM for 0 or 6 h were allowed to macropinocytose TRITC-dextran, and the median fluorescence intensity per cell of ingested TRITC-dextran were measured. The median fluorescence intensity per cell in WT cells starved for 0 h was considered 100%. (**B**) WT, *grlD^−^*, *ppk1^−^*, and *i6kA^−^* cells starved in PBM for 0 or 6 h were allowed to macropinocytose TRITC-dextran as in (**A**), uningested TRITC-dextran was removed, and cells were allowed to excrete the ingested TRITC-dextran for 1 h. The median fluorescence intensities per cell of TRITC-dextran retained after 1 h of excretion were determined. (**C**,**D**) WT, *grlD^−^*, *ppk1^−^*, and *i6kA^−^* cells were starved for 0 or 6 h and allowed to phagocytose Zymosan-A bioparticles for 1 h. The average median fluorescence intensity of ingested bioparticles, normalized to 0 h WT cells are shown in (**C**), and percentages of cells with ingested bioparticles are shown in (**D**). For all panels, cells were starved in stationary submerged culture. All values are mean ± SEM of 4 independent experiments. * indicates *p* < 0.05, ** *p* < 0.01, *** *p* < 0.001 (Two-way ANOVA with Šídák’s multiple comparisons test).

As previously observed [15,28], starvation for 6 h decreased macropinocytosis of TRITC-dextran in WT cells (Figure 4A). Starvation increased macropinocytosis in *grlD^−^* cells and had no significant effect on *ppk1^−^* and *i6kA^−^* cells (Figure 4A). Starvation reduces exocytosis [15], and in agreement with this we observed that after ingesting TRITC-dextran, and subsequently allowing 1 h for excretion of ingested TRITC-dextran, WT cells starved for 6 h retained more TRITC-dextran than freshly starved cells (Figure 4B). Starvation for 6 h did not significantly affect the retention of TRITC-dextran by *grlD^−^*, *ppk1^−^*, or *i6kA^−^* cells (Figure 4B). These results indicate that at 6 h after starvation, cells require GrlD, Ppk1, and I6kA to reduce macropinocytosis, decrease exocytosis, and increase retention of materials ingested by macropinocytosis.

### 2.5. Starving D. discoideum Cells Require GrlD, Ppk1, and I6kA to Reduce Phagocytosis

As with macropinocytosis (Figure 4A), and as previously observed [16], starvation for 6 h modestly reduced the net phagocytosis of yeast bioparticles in a population of WT cells (Figure 4C and Figure S1E). Analyzing the same data for the percentage of cells ingesting bioparticles, this was not accompanied by a reduced percentage of cells with phagocytosed yeast (Figure 4D and Figure S1E). Starvation for 6 h increased both phagocytosis and the percent of cells with phagocytosed yeast in *grlD^—^* and *ppk1^—^* cells, but had no significant effect in *i6kA^—^* cells (Figure 4C,D and Figure S1D–F).

### 2.6. Cells Lacking GrlD or Ppk1 Are Abnormally Large

**Figure 5:**
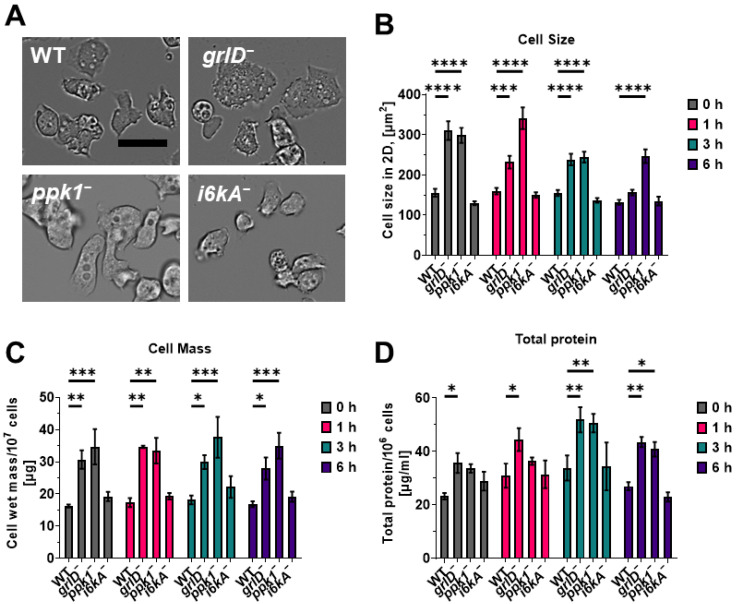
Starvation does not alter cell size, mass, and total protein content. (**A**) Representative images of vegetative WT, *grlD^−^*, *ppk1^−^*, and *i6kA^−^* cells growing in submerged stationary culture are shown from 3 independent experiments. Bar is 20 µm. (**B**) Cell areas of 10^6^ cells/mL WT, *grlD^−^*, *ppk1^−^*, and *i6kA^−^* cells starved in PBM in submerged stationary culture for 0, 1, 3, and 6 h. At least 50 cells from three independent experiments were taken to measure the cell size. (**C**,**D**) Cell mass per 10^7^ cells (**C**) and total protein content per 10^6^ cells (**D**) were determined for WT, *grlD^−^*, *ppk1^−^*, and *i6kA^−^* cells starved in PBM for indicated times. All values are mean ± SEM of 3 independent experiments. For (**B**,**D**), * indicates *p* < 0.05, ** *p* < 0.01, *** *p* < 0.001, and *p* < 0.0001 **** (Two-way ANOVA with Holm-Šídák’s multiple comparisons test).

At 6 h of starvation, *grlD^−^* and *ppk1^−^* cells had increased macropinocytosis and phagocytosis compared with WT cells, but no significant change in retention of ingested TRITC-dextran (Figure 4A–C). Although during starvation there is probably little nutrients for cells to uptake by phagocytosis, the increased macropinocytosis of *grlD^−^* and *ppk1^−^* cells might increase their mass. To examine this, WT, *grlD^−^*, *ppk1^−^*, and *i6kA^−^* were starved, and cell size, cell mass, and total protein content were measured. Although *grlD^−^* and *ppk1^−^* cells tended to be larger and have more mass than WT cells (Figure 5A–C), their mass did not significantly increase from 0 to 6 h compared to WT cells, suggesting that the increased macropinocytosis of *grlD^−^* and *ppk1^−^* cells did not cause them to significantly gain additional mass over the first 6 h of starvation. Compared with WT cells, *grlD^−^* cells had more total protein content at all times examined, probably because they are intrinsically larger cells, and *ppk1^−^* cells had more protein at 3 and 6 h of starvation (Figure 5D), suggesting that for unknown reasons, they convert nutrient stores to protein.

## 3. Discussion

PolyP is an autocrine signaling molecule in *D. discoideum*. Cells growing in the presence of nutrients (vegetative cells) secrete polyP, and at high cell densities where the cells are about to overgrow their food source, the concomitant high extracellular concentrations of polyP inhibit cell proliferation without compromising cell growth [27]. PolyP also primes cells to anticipate starvation and prepare them to enter starvation-induced development [24]. Here, we showed that starved *D. discoideum* cells accumulate soluble and cell surface polyP and can bind exogenous polyP. PolyP inhibits the membrane fluidity of vegetative cells [28], and here we observed that compared to vegetative cells, starved cells have a decreased membrane fluidity.

Ppk1 synthesizes polyP, and as expected cells lacking Ppk1 have reduced accumulation of both extracellular and cell surface polyP during starvation. PolyP decreases membrane fluidity, and so also as expected, *ppk1^−^* cells do not decrease membrane fluidity during starvation. Supporting the idea that polyP mediates the starvation-induced decrease in macropinocytosis, phagocytosis, and exocytosis, *ppk1^−^* cells do not exhibit these decreases. Compared to WT cells, *ppk1^−^* cells have reduced binding of exogenous polyP, suggesting the possibility that polyP increases levels of the receptor that binds polyP.

GrlD is the receptor that binds and senses polyP, and as expected starved *grlD^−^* cells show reduced binding of exogenous polyP. Unexpectedly, the *grlD^−^* cells do not accumulate either extracellular or cell-surface polyP, suggesting that there exists some sort of feedback mechanism where sensing some polyP is needed to accumulate extracellular polyP. This might prevent cells from wasting energy releasing polyP in a situation where the cell is in an environment such as a rainstorm where the polyP will be immediately washed away. As a result of either not sensing or not accumulating extracellular polyP, cells lacking GrlD do not decrease membrane fluidity, macropinocytosis, phagocytosis, and exocytosis during starvation, further supporting the idea that sensing extracellular polyP mediates these decreases.

Along with Ppk1, I6kA is involved in the accumulation of polyP in vegetative cells, and in starved *i6kA^−^* cells we observed normal levels of extracellular polyP but reduced levels of cell surface polyP. An intriguing possibility is that I6kA generates a polyP that has an inositol at one end, and that the cell surface polyP is associated with this hypothetical modified polyP. The *i6kA^−^* cells show reduced binding of exogenous polyP, suggesting that I6kA affects the GrlD receptor directly, or that cell-surface polyP is needed for GrlD localization to the cell surface, or that cell-surface polyP is needed for GrlD function. Cells lacking I6kA show a normal decrease in membrane fluidity at 6 h of starvation, but do not decrease macropinocytosis, phagocytosis, and exocytosis. This then indicates that during starvation, the increase in cell surface polyP is not responsible for the decrease in membrane fluidity, and that reduced cell surface polyP but not altered membrane fluidity is not responsible for the starvation effects on macropinocytosis, phagocytosis, and exocytosis.

High levels of polyP increase the size and mass of vegetative cells [27]. This would initially suggest that *grlD^−^* cells that do not sense polyP [32], and *ppk1^−^* cells, which make no detectable intracellular polyP [30] and have ~a 50% reduction in extracellular polyP [27], would tend to be small. However, we observed that vegetative and starved *grlD^−^* and *ppk1^−^* cells are abnormally large. Both *grlD*^−^ and *ppk1*^−^ cells are defective in cytokinesis and are multinucleate, which can cause cells to become abnormally large [29,39]. For the *grlD^−^* cells, one possible explanation is these cells sense that there is an abnormally low level of extracellular polyP, and as a result the *grlD^−^* cells inappropriately sense that they have become isolated from a colony of other cells, and decrease cytokinesis and thus increase cell size and nutrient reserves in response to this abnormal situation. For the *ppk1^−^* cells, one possible explanation is that making polyP is energetically costly, and not making intracellular polyP allows the *ppk1^−^* cells to be larger than WT cells; another possibility is that polyP helps cytokinesis and thus there is defective cytokinesis in the *ppk1^−^* cells. Together, these results suggest that in addition to acting as a cell density sensing signal during growth, polyP has significant functions as a signal during development.

## 4. Materials and Methods

### 4.1. D. discoideum Cell Culture

WT AX2 (DBS0237699) [4], *grlD^−^* (DBS0350227) [39], *ppk1^−^* (DBS0350686) [30], and *i6kA^−^* (DBS0236426) [40] *D. discoideum* strains were obtained from the *Dictyostelium* Stock Center [41]. *D. discoideum* cell cultures were maintained at 21 °C in type 353003 tissue culture dishes (Corning, Durham, NC, USA) in 10 mL of SIH defined minimal medium (Formedium, Norfolk, UK) under no selection (AX2) or selection with 5 µg/mL blasticidin (*grlD^−^*, *ppk1^−^*, and *i6kA^−^*). Cells were also grown on SM/5 agar [42] on lawns of *E. coli* DB (DBS0350636) in a type 25384-302 Petri dish (VWR, Radnor, PA). 100 µg/mL dihydrostreptomycin and 100 µg/mL ampicillin were used to kill *E. coli* in *D. discoideum* cultures obtained from SM/5 agar [43]. *D. discoideum* cells from 80–90% confluent cultures in a tissue culture dish were collected using a sterile glass pipette, transferred to 15 mL conical tubes (Falcon, VWR), washed 2 times with SIH by centrifugation at 500× *g* for 5 min, the cell density was measured with a hemocytometer, and 300 µL of cells at 1 × 10^6^ cells/mL was transferred to the well of a type 353219 96-well, black/clear, tissue culture treated plate (Corning) to obtain 3 × 10^5^ cells per well, or 1 mL was transferred to the well of a type 353047 24-well tissue culture plate (Corning) to obtain 10^6^ cells per well. For starvation assays, *D. discoideum* cells in a 96-well, black/clear, tissue culture treated plate or a 24-well tissue culture plate were washed twice with PBM (20 mM KH_2_PO_4_, 0.01 mM CaCl_2_, and 1 mM MgCl_2_, pH adjusted to 6.1 with KOH) by centrifuging the plate at 500× *g* for 3 min and replacing the supernatant with fresh PBM, and cells were incubated for 0, 1, 3, and 6 h for starvation. The 24- and 96-well plates with cells were incubated in a Tupperware container with wet paper towels for humidity.

### 4.2. Recombinant Polyphosphatase Purification and polyP Concentration Measurement

Recombinant *Saccharomyces cerevisiae* exopolyphosphatase (PPX) [36,44] was purified following the method described for the purification of recombinant autocrine proliferation repressor protein AprA [45]. *D. discoideum* cells and culture supernatants were treated with PPX as previously described [46]. PolyP concentrations were determined in conditioned medium (CM) following [28]. Briefly, cultures of cells growing in SIH, or starved in PBM for the indicated times (both in stationary submerged culture as described above) were gently swirled and the supernatant was collected. This was then clarified by centrifugation at 300× *g* for 5 min. The supernatant was then clarified by centrifugation at 12,000× *g* for 2 min, and the supernatant was collected and designated CM. The CM was incubated with 25 µg/mL DAPI (Biolegend, San Diego, CA, USA) for 5 min at room temperature, and fluorescence was measured at 550 nm after exciting at 415 nm. PolyP standards (Spectrum, Cat# S0169; New Brunswick, NJ, USA) were used to determine the concentration of polyP in the CMs.

### 4.3. PolyP Binding Assay and SYTOX Staining of Membrane-Bound polyP

PolyP binding assays were performed as previously described [32], except that tag-free polyP was utilized instead of biotinylated polyP, and the bound polyP was stained with the polyP-binding fluorescent dye SYTOX as previously described [35]. Briefly, *D. discoideum* cells in a 96-well, black/clear, tissue culture-treated plate were spun down at 500× *g* for 3 min. The SIH medium was replaced with fresh SIH medium containing 705 µg/mL polyP and incubated for 3 min. Cells were spun down at 500× *g* for 3 min and the medium was replaced with fresh SIH. This step was repeated twice to remove unbound polyP. SIH medium containing 1.5 µM SYTOX (Cat#S7020, Thermo Fisher Scientific, Waltham, MA, USA) was added to the cells and incubated for 10 min, and images were taken using a 40× objective on a Nikon Eclipse Ti2 inverted microscope (Nikon, Melville, NY, USA). Deconvolution of images was carried out using the Richardson-Lucy algorithm [47] in Nikon NIS-Elements AR software. The fluorescence intensity of SYTOX staining was analyzed by ImageJ [48]. SYTOX staining of cells in SIH or cells starved in PBM for 2, 3, or 6 h was performed as described above but in the absence of added exogenous polyP. Where indicated, 5 µg/mL PPX was added to the cells incubated in the PBM during the 2, 3, or 6 h of starvation.

### 4.4. DNase and RNase Treatments

Cells were starved in PBM for 5.5 h in a 96-well, black/clear, tissue culture-treated plate as described above and 50 µg/mL RNase (Cat#109142, Roche CustomBiotech, Indianapolis, IN, USA) or 50 µg/mL DNase (04536282001, Roche Diagnostics, Mannheim, Germany) were added to cells, and the cells were incubated for 30 min, SYTOX staining of the cells was performed, and images of the cells were taken as described above. To test the enzymatic activity of RNase and DNase, 1 µg of total RNA isolated from WT *D. discoideum* cells as previously described [49] or 1 µg of a plasmid DNA PDM232 [50] was incubated without or with 50 µg/mL of RNase or DNase, respectively, for 45 min at room temperature, and resolved by 0.7% agarose gel electrophoresis with ethidium bromide stain [51].

### 4.5. Fluorescence Recovery after Photobleaching (FRAP)

Photobleaching assays to measure the cell membrane fluidity were performed as previously described [28,52], except that the *D. discoideum* cells were starved in PBM buffer for the indicated times.

### 4.6. Macropinocytosis and Exocytosis Assays

For both macropinocytosis and exocytosis, tetramethylrhodamine isothiocyanate–dextran (TRITC-dextran) (T1162-100MG, Sigma-Aldrich, St. Louis, MO. USA) was used to visualize ingestion and retention in adhered *D. discoideum* cells [28,53]. For macropinocytosis, cells were starved in a 24-well plate in PBM with 5 µL of 1 mg/mL TRITC-dextran for 1 h to allow macropinocytosis of the TRITC-dextran. After 1 h, the cells incubated with TRITC-dextran were spun down at 500× *g* for 3 min, the supernatant was replaced with fresh PBM, and this step was repeated twice. Cells were gently washed off of the bottom of the plate with 200 µL PBM. The median fluorescence of the live cell population was recorded using the PE-A fluorescence gate on a Accuri C6 flow cytometer (BD, San Jose, CA, USA). The same procedure was repeated for the 6 h starved cells by adding TRITC-dextran at 6 h of starvation and collecting the cells at 7 h.

The exocytosis assay was performed similarly to the endocytosis assay. Freshly starved cells were incubated with TRITC-dextran for 1 h, and the cells were washed 3 times with PBM as above, an aliquot of cells was collected and the fluorescence of the retained ingested TRITC-dextran was measured using the flow cytometer, and 1 mL of PBM was then added to the remaining cells to allow exocytosis. After 1 h, cells were collected, and the fluorescence of the retained ingested TRITC-dextran was measured. Similarly, cells starved for 6 h were incubated with TRITC-dextran for 1 h, and the cells were washed as above with PBM, and 1 mL of conditioned medium collected from cells starved for 6 h in PBM in a separate culture were added to the cells with ingested TRITC-dextran to allow exocytosis for 1 h. The percentage of TRITC-dextran retained after exocytosis was calculated from the median fluorescence of the ingested TRITC-dextran in the cells that were allowed to exocytose for 1 h divided by the median fluorescence of the ingested TRITC-dextran in the cells that were not allowed to exocytose.

### 4.7. Phagocytosis Assay

*D. discoideum* cells were starved for 0 and 6 h, as described above. The cells were collected and 800 µL was used to measure background fluorescence intensity in the PerCP-A channel in the Accuri C6 flow cytometer. The remaining 200 µL in the wells were incubated with 5 µL of 1 mg/mL of Alexa 594-labeled Zymosan-A yeast BioParticles (Cat#Z23374, Thermo Fisher Scientific, Waltham, MA, USA) in PBM and allowed to phagocytose for 1 h. Cells were collected, and the fluorescence of the live cell population was measured using the fluorescence gating for Alexa 594 on the flow cytometer (Supplementary Figure S1). *D. discoideum* cells were identified and gated by their size and granularity as indicated by P7 in Supplementary Figure S1A. *D. discoideum* cells without phagocytosed yeast, only yeast, or *D. discoideum* cells with phagocytosed yeast were identified by their size and fluorescence (Supplementary Figure S1B–D). This generated the graphs showing a histogram of fluorescence intensity on the *x*-axis and number of events (cells) on the *y*-axis (Supplementary Figure S1E–H). Using the Alexa Fluor 594 fluorescence channel, a linear marker gate was drawn to encompass 100% of the cells in the histogram. The fluorescence intensities of the *D. discoideum* cells with phagocytosed yeast were then analyzed for the median fluorescence intensities, which were then normalized to the median fluorescence intensities of the 0 hour starved WT *D. discoideum* cells with the phagocytosed yeast (Figure 4C).The percentages of *D. discoideum* cells with phagocytosed yeast were also analyzed from the histograms by counting the number of cells in the high fluorescence peak area (bars in Supplementary Figure S1E–H) as a percentage of the total number of cells.

### 4.8. Cell Size, Mass, and Protein Determination

To determine cell size, mass, and total protein content, 50 mL of cells at 1 × 10^6^ cells/mL were starved as described above in a shaking culture for 0, 1, 3, or 6 h. For cell size measurement, at each time point, 100 µL of cells were transferred to a 96-well, black/clear, tissue-culture-treated plate, and images of cells were taken using a 40× objective on a Nikon Eclipse Ti2 inverted microscope. Cell sizes were measured using Fiji (ImageJ; NIH, Bethesda, MD, USA). At least 50 cells from each of three independent experiments were used to measure cell size.

For cell mass and total protein content measurement, cells from 10 mL starved cells were collected by centrifugation at 500× *g* for 3 min. The pellet was resuspended in approximately 500 µL of residual PBM and transferred to microcentrifuge tubes. The cells were collected by centrifugation at 3000× *g* for 3 min. The supernatant was discarded, and the cell pellets were weighed. The total protein content of cells was determined as previously described [54].

### 4.9. Statistical Analysis

Statistical analysis was performed using Prism 9 (GraphPad, San Diego, CA, USA) and the tests indicated in the figure legends. A *p* < 0.05 was considered to be significant.

## Data Availability

Further information and requests for reagents may be directed to, and will be fulfilled by, the authors Ramesh Rijal (rijalramesh@tamu.edu) and Richard Gomer (rgomer@tamu.edu).

## Supplementary Materials

The following supporting information can be downloaded at: https://www.mdpi.com/article/10.3390/ijms24065923/s1, Figure S1: Starving *D. discoideum* cells require GrlD, Ppk1, and I6kA to reduce phagocytosis.
