# Supplementary material for: Starvation Induces Extracellular Accumulation of Polyphosphate in Dictyostelium discoideum to Inhibit Macropinocytosis, Phagocytosis, and Exocytosis
Source: Int J Mol Sci. 2023 Mar 21;24(6):5923. doi: 10.3390/ijms24065923 (PMC10056890; doi:10.3390/ijms24065923)
Supplement: Supplementary file 1 [file ijms-24-05923-s001.zip › ijms-2255400-supplementary.pdf]

Supplementary Figure S1

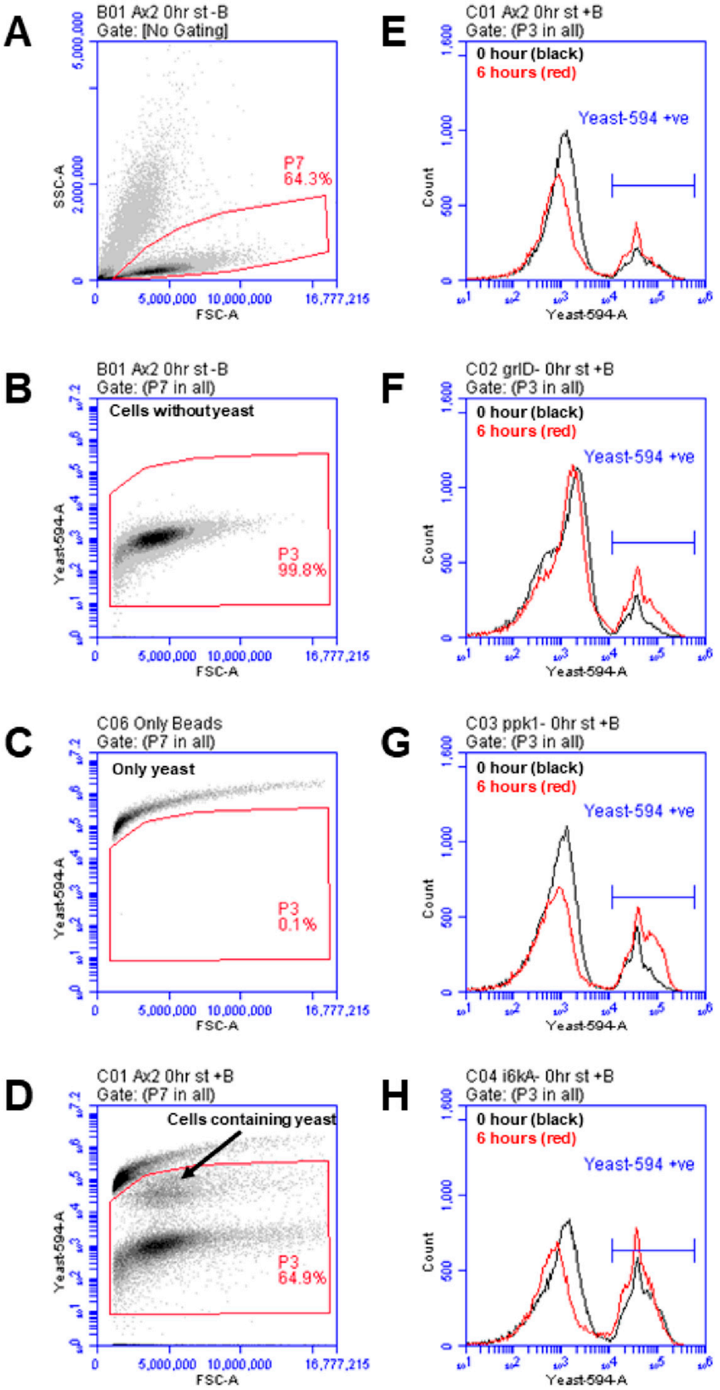

Figure S1. Starving *D. discoideum* cells require GrlD, Ppk1, and I6kA to reduce phagocytosis. WT, *grlD*$^-$, *ppk1*$^-$, and *i6kA*$^-$ cells starved in PBM for 0 or 6 hours were allowed to phagocytose Alexa 594-labeled Zymosan-A bioparticles for 1 hour and prepared for flow cytometry. Cells were identified by their size

(FSC-A) and granularity (SSC-A). **A** shows the forward scatter (X-axis) and side scatter (Y-axis), and the red line delineates the region (P7) used to gate *D. discoideum* cells. Panels **B-D** show forward scatter (X-axis) and fluorescence (Y-axis) of (**B**) cells only, (**C**) Zymosan-A bioparticles (yeast) only, and (**D**) cells with phagocytosed yeast. **E-H**) The fluorescence intensity of ingested bioparticles in 0 (black) and 6 (red) hours starved (**E**) WT, (**F**) *grlD*$^{-}$, (**G**) *ppk1*$^{-}$, and (**H**) *i6kA*$^{-}$ *D. discoideum* cells. Bars in E-H designate the high-fluorescence cells (and thus the cells containing Alexa 594-labeled Zymosan-A bioparticles). Plots are representative of cells from 4 independent experiments.
